# Repeated Detection of *Bartonella* DNA in Feline Placenta: Potential Implications for Placental and Fetal Development

**DOI:** 10.3390/ani15142041

**Published:** 2025-07-11

**Authors:** Charlotte O. Moore, Ricardo Maggi, Kelli Ferris, Edward B. Breitschwerdt

**Affiliations:** 1 Intracellular Pathogens Research Laboratory, Department of Clinical Sciences, College of Veterinary Medicine, North Carolina State University, Raleigh, NC 27607, USA; comanvel@gmail.com (C.O.M.); rgmaggi@ncsu.edu (R.M.); 2 Department of Clinical Sciences, College of Veterinary Medicine, North Carolina State University, Raleigh, NC 27607, USA

**Keywords:** *Bartonella*, cat, vertical transmission, placenta

## Abstract

The domestic cat is the primary reservoir host of three flea-borne *Bartonella* species, one of which (*Bartonella henselae*) causes reduced fertility and reproductive failure in experimentally infected cats. Vertical transmission of *Bartonella* has been documented only in B-cell deficient mice, but not immunocompetent animals. As many free-roaming cats are chronically infected with *Bartonella* and may be immunocompromised by environmental stress or coinfection, we attempted to isolate *Bartonella* from the fetal and placental tissues of pregnant queens spayed during trap–neuter–release. Four samples from each tissue (ovary, uterus, fetus, and placenta) were split for direct DNA extraction, liquid culture, and culture on a blood agar plate. Samples from infected queens were inoculated into liquid media and sampled weekly for three weeks for DNA extraction and plating. *Bartonella* DNA was sequenced directly from 28% (5/18) of the free-roaming queens. For these five queens, liquid enrichment culture was attempted in duplicate for fetal and placental samples. *Bartonella clarridgeiae* DNA was amplified using qPCR liquid enrichment cultures from the placentas of two cats. These findings suggest that viable *Bartonella* organisms are present in feline reproductive tissue. Additional studies are needed to assess the transplacental transmission of *Bartonella* spp. and *Bartonella*’s influence on fetal development.

## Simple Summary

Three *Bartonella* species commonly infect the domestic cat, and may make it difficult for cats to become pregnant or have healthy offspring. The transmission of *Bartonella* from cats to kittens does not occur in healthy cats, but free-roaming domestic cats are often chronically stressed and/or co-infected with other pathogens. Therefore, we tested the placenta, fetus, ovary, and uterus tissue of free-roaming domestic cats to detect Bartonella DNA or culture live *Bartonella. Bartonella* DNA was detected in 28% (5/18) cats. *Bartonella* was not grown from any cat, but *Bartonella* DNA was detected in liquid culture. Therefore, we concluded that viable *Bartonella* may infect the cat’s placenta. Further research is necessary to understand if *Bartonella* can infect the placenta or be transmitted from cats to kittens.

## 1. Introduction

The domestic cat is the primary reservoir host of three zoonotic *Bartonella* species (*Bartonella clarridgeiae*, *Bartonella henselae*, and *Bartonella koehlerae*) that are transmitted by the cat flea (*Ctenocephalides felis*). With a worldwide distribution, the cat and cat flea are ubiquitous in nearly all areas populated by human beings. *Bartonella clarridgeiae* is the most common species infecting cats and fleas, while *B. henselae* and *B. koehlerae* are more pathogenic in cats and humans [1,2]. Due to the difficulty of achieving a diagnosis of bartonellosis by culture isolation or PCR amplification of *Bartonella* spp. DNA, particularly in non-reservoir adapted hosts, studies involving naturally infected cats can provide insights into disease manifestations associated with infection. *Bartonella* DNA has been amplified from the female reproductive tissues of multiple species; however, its potential pathogenic effects remain unclear [3,4].

Current knowledge regarding the effect of *Bartonella* spp. infection on reproductive performance are based upon experimental infections and naturally infected animals or humans. Experimental infection of five queens with culture-grown *B. henselae* documented infertility upon repeated breeding attempts [5]. In the three cats that conceived, one kitten was born with hydrocephalus, and one was stillborn. In contrast, the uninfected control cat, bred by the same sire, became pregnant by the first breeding and delivered healthy kittens. Experimental infection of mice with *Bartonella birtlesii*, a species that infects mice and rats in nature, similarly documented increased fetal death and resorption and decreased fetal weight [6]. In mice experimentally infected with *Bartonella taylorii*, vertical transmission occurs only in the absence of B-cell adaptive immunity [7]. This finding in mice is reinforced by the lack of documented *B. henselae* vertical transmission in healthy, experimentally infected domestic cats [5,8]. These young, specific-pathogen-free cats, maintained in environmentally controlled facilities and feed an optimal diet, do not represent the health status of free-roaming cats with limited shelter and food. In addition, approximately 7% of feral cats are infected with FIV or FeLV, immunosuppressive retroviruses [9].

In the context of natural infection, multiple species of wild-caught voles (*Microtus* spp.) transmit *Bartonella* spp. to their offspring in a vector-free environment, with over half of offspring infected at 3 weeks of age [10]. Similar findings have been documented in smaller surveys of wild-caught rodents *Peromyscus leucopus* and *Sigmodon hispidus* [11]. *Bartonella bovis* infection in dairy cows was associated with a shorter interval from calving to artificial insemination and decreased incidence of placental retention, but other influences on bovine reproduction were not observed [12]. The limited associations of bartonellosis and reproduction to date supports the need to explore the effect of *Bartonella* spp. on reproduction in humans and other domestic and wildlife animal species. Prior documentation of *B. henselae* DNA in the placenta and fetal tissues of free-roaming cats suggests that the pathogen can circulate to the fetus [3]. To further assess the potential for placental or fetal *Bartonella* infection, we attempted to detect pathogen DNA via qPCR and to isolate *Bartonella* spp. from the fetus and placental tissues from naturally infected cats utilizing 21-day liquid enrichment culture and blood agar plating.

## 2. Materials and Methods

Reproductive tracts were obtained under NCSU Institutional Care and Use Committee protocol #21-468 via ovariohysterectomy from 18 free-roaming domestic cats during a trap-neuter-release program to assist in the control of free-roaming cat populations.

Reproductive tracts were frozen and transferred to the NCSU Intracellular Pathogens Research Laboratory for testing. Prior to dissection, tracts were thawed at 4 °C for two days. Tissue storage tubes were autoclaved. With a sterile scalpel, two ovary and two uterus samples were collected. After the scalpel and forceps were sterilized, the uterus was incised to reveal the placenta and amnionic sac, and scalpel and forceps were again sterilized. Placental samples were removed and the amnionic sac was lifted above a sterile Petri dish and cut to allow the fetus to drop into the dish. The scalpel and forceps were sterilized again. Total fetal length was measured crown to rump. Depending on the stage of gestation, fetal samples included the whole fetus or primarily abdominal organs. After sterilization of the scalpel and forceps, the procedure was repeated for the second fetus. All ovary, uterus, placental, and fetal samples were collected in duplicate.

Each tissue was individually bead beat (Fisherbrand™ Bead Mill 4 Mini Homogenizer, Fisher Scientific, Waltham, MA 02451, USA) with three metal beads until fully pulverized, and then inoculated with 1 mL of Brugge liquid enrichment culture media [13]. Bead beating controls were generated by bead-beating in an empty tube. Following Brugge inoculation, 200 µL was immediately removed for direct DNA extraction (Qaigen DNeasy Blood & Tissue Kit, Qiagen, Valencia, CA, USA) and *Bartonella* qPCR for the *ssrA* gene [14]. Fetal and placental samples from cats with ≥1 tissue qPCR positive for *Bartonella* DNA were selected for culture. For these samples, 200 µL of the original tissue and Brugge mixture was inoculated onto a blood agar plate (TSA with 5% sheep blood, ThermoFisher Scientific, Waltham, MA, USA). An additional 600 µL was inoculated into 10 mL of Brugge and 1 mL of defibrinated sheep’s blood (Carolina Biological Supply, Burlington, NC, USA). At 7, 14, and 21 days, 200 µL of the liquid enrichment culture was sampled for DNA extraction and qPCR, and 500 µL of liquid culture was inoculated onto a blood agar plate [13]. Blood agar plates and liquid culture were incubated at 37 °C and 5% CO_2_. Blood agar plates were kept for at least 6 weeks. Liquid culture negative controls consisted of Brugge culture medium subjected to bead-beating and liquid culture alone.

Based on the lack of successful blood agar plate isolation, frozen (−20 °C) stored tissues were utilized to reattempt culture isolation following incubation with Brugge (cat R4 and R7) or BAPGM (cat R9, R14, and R21) media [15]. BAPGM media culture was selected for R9, R14, and R21 to increase the likelihood of successful *Bartonella* isolation, as Brugge re-isolation of R4 and R7 had already been attempted and did not yield a *Bartonella* isolate. Tissues cultured in Brugge were performed as described above, while tissues cultured in BAPGM were inoculated into a T12.5 flask with 10 mL of BAPGM and 1 mL of defibrinated sheep’s blood.

## 3. Results

Reproductive tracts were obtained from 18 pregnant free-roaming cats. *Bartonella* spp. DNA was amplified from tissue extractions in five queens (5/18, 28%; Table 1). *Bartonella* DNA was amplified and sequenced from all sample types: ovary (11%, 4/36), placenta (8%, 3/36), fetus (2/36, 5.5%), and uterus (2/36, 5.5%). Three queens were infected with *B. henselae*, one was infected with *Bartonella clarridgeiae*, and one was infected with *Bartonella vinsonii* subsp. *berkhoffii.* Average fetal length (crown-rump) was 7.7 cm, but varied greatly with a minimum of 1.3 cm and maximum of 14.3 cm. Fetal length was similar in *Bartonella* DNA detected (7.17 ± 1.48 cm, mean ± SEM) and no *Bartonella* DNA detected (7.96 ± 0.98 cm) queens (*p* = 0.67).

Following liquid culture for 7, 14, and 21 days of incubation, *Bartonella* spp. DNA was amplified and sequenced from placental enrichment culture DNA extractions from two cats (Table 2). Infection with *B. clarridgeiae* was again documented using multiple enrichment culture timepoints in cat R7, in conjunction with co-infection with *Bartonella koehlerae*. Based upon direct tissue extraction and enrichment culture, cat R14 was co-infected with *B. vinsonii* subsp. *berkhoffii* and *B. clarridgeiae*.

Other fetal and placental enrichment culture samples were all qPCR-negative, and subculturing onto blood agar plates was not successful for any fetal or placental sample. Negative media and bead-beating controls remained qPCR-negative throughout the study period.

## 4. Discussion

This study documents the molecular presence of four *Bartonella* species in reproductive tissues of domestic free-roaming cats, including the detection of *B. clarridgeiae* DNA in ovary, uterus, fetus, and placental tissues. In addition, coinfection with two *Bartonella* spp. was documented in two out of five infected cats. As agar plate isolation was not successful for any tissue or at any enrichment subculture time point, documentation of *Bartonella* DNA in liquid culture supported bacterial growth, but did not definitively confirm bacterial viability. It is important to emphasize that even in tissues that are known to be *Bartonella*-infected, direct isolation or isolation with liquid subculture displays limited sensitivity. In R7 (a *B. clarridgeiae* infected cat), *B. clarridgeiae* was detected in all tissues using direct DNA extraction, as well as the placenta at all liquid culture days (7, 14, and 21 days); however, *B. koehlerae* coinfection was only detected in Brugge placental culture after 21 days of incubation. *Bartonella clarridgeiae* and *B. henselae* are detected in flea and cat samples more often than *B. koehlerae*, which, combined with our detection only at 21 days of liquid culture, suggests that diagnosis of *B. koehlerae* may be limited by low bacteremia or slower growth than other *Bartonella* species [1].

As with other mammalian species, the feline placenta is highly perfused with blood. Therefore, amplification of *Bartonella* spp. DNA may represent bacteria within the maternal blood supply or bacteria that have invaded and colonized placental tissues. As with the induction of placental lesions in mice experimentally infected with *B. birtlesii*, it is possible that *Bartonella* infection has a pathogenic effect on the placenta of the domestic cat [6]. Further research is necessary to determine if the reproductive failure of cats experimentally infected with *B. henselae*, as reported by Guptill and colleagues, may be attributed to pathological changes in placentation or fetal infection [5]. The effect of chronic bloodstream *Bartonella* infection on fetal weight, fetal resorption, and fetal developmental defects, the negative impacts of which were reported in *B. birtlesii* infected female mice, should also be explored in mothers, their progeny, and domestic and wildlife animal species [6]. Interestingly, *Brucella*, another alphaproteobacterial closely related to *Bartonella*, is a well-recognized cause of abortion and reproductive failure in multiple animal species [16].

The potential for vertical transmission of *Bartonella* spp. also has important implications for future disease manifestations in offspring and maintenance of these bacteria within various environments. Our inability to isolate *Bartonella* was expected, as *Bartonella* can be very difficult to isolate onto blood agar plates from chronically infected animal and human patients. The transition from liquid media (BAPGM or Brugge) to growth on a blood agar plate appears to be a rate-limiting step in the isolation process. In chronically infected cats or humans, *Bartonella* displays low organism numbers and a relapsing bacteremia, resulting in manuscripts utilizing multiple diagnostic methods to increase sensitivity [17,18,19]. The repeated documentation of *B. clarridgeiae* DNA at 7, 14, and 21 days of BAPGM enrichment cultures supports, but did not confirm, the presence of viable bacteria.

Free-roaming kittens display high mortality rates due to traumatic and infectious causes [20]. After experimental infection of B-cell deficient female mice, *B. taylorii* was repeatedly successfully isolated from offspring at 4–5 weeks [7]. The potential for stress, co-infection, chronic infection, or immunosuppression (e.g., retroviral infection, immunosuppressive drugs) to permit vertical transmission in mice, cats, or humans is unknown. The medical, microbiological, and pathological complexity of these and other factors illustrates the need for prospective, targeted, and structured studies to investigate the role of this genus in reproductive failures among animal species. Results that can be derived from studying *Bartonella* chronically infected free-roaming cats that are at high risk for FIV, FeLV, and other infections may provide a basis for comparative medical investigations.

In 2009, histologic, immunohistochemical, ultrastructural, and molecular methods were used to confirm *B. henselae* infection in an aborted equine fetus, providing the first evidence of reproductive failure in a non-reservoir host [21]. Case studies or case series also suggest in utero or perinatal transmission of *Bartonella* in humans. One case study documented perinatal transmission of *B. henselae* and *B. vinsonii* subsp. *berkhoffii* from the mother to her child, who died of a congenital heart defect at 9 days of age [4]. The twin brother and mother were *B. henselae* and *B. vinsonii* subsp. *berkhoffii* bacteremic when tested ten years after parturition, supporting the potential for chronic intravascular infection spanning a decade. As both *B. henselae* and *B. vinsonii* subsp. *berkhoffii* DNA were amplified and sequenced from the tissues of the child who died due to a congenital heart defect, a potential role for this genus should be investigated as a cause of abnormal embryogenesis [4]. In a small case series from Israel involving eight pregnant women diagnosed with Cat Scratch Disease, caused by *B. henselae* and potentially *B. clarridgeiae*, one woman had a spontaneous abortion, one terminated the pregnancy, and the remaining six had healthy newborns, with no reported sequalae for a median 4.5-year follow-up period [22]. Potential in utero infection with *B. henselae* was suspected in a 14-year-old boy with congenital cerebellar hypoplasia, who subsequently developed progressive, fluctuating neuropsychiatric symptoms [23]. Another case study reported the intrauterine fetal demise and assisted delivery at 31-week gestation from a 24-year-old mother presenting with relapsing low-grade fever and malaise for the previous month [24]. At this time, infection was not suspected and, therefore, additional microbiological investigation not pursued. Ultrasonography and computed tomography (CT) revealed a heteroechoic lesion in segment VI and VII of the liver in the mother. The mother subsequently underwent a right hepatectomy, which enabled the practitioners to discover an exophytic solid-cystic mass in segment VI and VII of the liver, a ruptured liver capsule, and intralesional hematoma, which were surgically resected. Histopathology, serology, and PCR results supported a diagnosis of *B. henselae* infection, with no prior reported cat contact.

The molecular detection of *Bartonella* coinfections in two queens only after testing more than one tissue using enrichment culture followed by DNA extraction and PCR testing further illustrates the limitations of current microbiological techniques for confirming or excluding infection with these bacteria. In a recent case report, infection with *B. henselae* was confirmed by enrichment brain biopsy culture in both BAPGM and Brugge media six years after the child was scratched on the face by a free-roaming cat [25]. Prior *Bartonella* spp. serology and enrichment culture of blood were both negative, illustrating the low-level chronic infection induced by these bacteria and the importance of tissue specific analyses in assessing whether an individual is or is not infected. Co-infection with multiple *Bartonella* species or other protozoa and bacterial species is widely reported in multiple animal species, including cats, dogs, and humans [3,26,27,28].

## 5. Conclusions

In conclusion, we documented the presence of *Bartonella* spp. DNA in ovary, uterus, placenta, and fetal tissues obtained from pregnant domestic free-roaming cats. Repeated enrichment culture of *B. clarridgeiae* from placental tissue of one cat supported the possibility of intrauterine infection with viable bacteria. Exploring the pathology of *Bartonella* infection in cat uterus, placental, and fetal tissues may identify previously unrecognized manifestations, with implications for zoonotic infection or transmission. The potential for vertical transmission in chronically infected mammalian reservoir and non-reservoir species remains unclear, and it is likely influenced by the infecting *Bartonella* spp., the evolutionary adaptation of the reservoir host, and the immune competence of the incidental host.

## Figures and Tables

**Table 1:** PCR amplification of the *Bartonella ssrA* gene from direct reproductive tissue extractions.

Queen Number	Fetal Length (cm)	Ovary	Uterus	Placenta	Fetus
R4	7	1/2—*B. henselae* (MK298190.1, 164/165 bp)	0/2	0/2	0/2
R7	11.5	1/2—*B. clarridgeiae* (MK298175.1, 159/159 bp)	1/2—*B. clarridgeiae* (MK298175.1, 159/159 bp)	1/2—*B. clarridgeiae* (93/93, MN809215.1)	1/2—*B. clarridgeiae* (MK298175.1, 159/159 bp)
R9	6.5	0/2	1/2—*B. henselae* (MK298190.1, 156/157 bp)	0/2	0/2
R14	2.4	1/2—*B. vinsonii* subsp. *berkhoffii* (MG432827.1, 149/150)	0/2	0/2	0/2
R21	8.5	1/2—*B. henselae* (MK298190.1, 158/158 bp)	0/2	0/2	0/2

Number of qPCR-positive samples from each tissue and *Bartonella* spp. determined using NCBI BLAST comparison (blast.ncbi.nlm.nih.gov, accessed on 4 July 2025). Negative samples were omitted for readability. Fetal length is expressed as the average of two fetuses in centimeters.

**Table 2:** Detection of *Bartonella* DNA via qPCR amplification and sequencing from a liquid culture medium at multiple time points.

Queen Number	Tissue	Culture Medium	Culture Day	*Bartonella* spp.
R7	Placenta	Brugge (first culture)	7	*B. clarridgeiae* (MK298175.1, 159/159 bp)
R7	Placenta	Brugge (first culture)	14	*B. clarridgeiae* (MK298175.1, 159/159 bp)
R7	Placenta	Brugge (first culture)	21	*B. clarridgeiae* (MK298175.1, 159/159 bp)
R7	Placenta	Brugge (second culture)	21	*B. koehlerae* (JN029769.1, 166/167 bp)
R14	Placenta	Brugge (second culture)	14	*B. clarridgeiae* (MK298175.1, 165/168 bp)

## Data Availability

The original contributions presented in this study are included in the article. Further inquiries can be directed to the corresponding author(s).
